# Genome Sequences for Two Acinetobacter baumannii Strains Obtained Using the Unicycler Hybrid Assembly Pipeline

**DOI:** 10.1128/MRA.00017-21

**Published:** 2021-03-11

**Authors:** John M. Farrow, Everett C. Pesci, Daniel J. Slade

**Affiliations:** a Department of Microbiology and Immunology, The Brody School of Medicine at East Carolina University, Greenville, North Carolina, USA; b Department of Biochemistry, Virginia Polytechnic Institute and State University, Blacksburg, Virginia, USA; Indiana University, Bloomington

## Abstract

Here, we report a complete genome sequence for Acinetobacter baumannii strain ATCC 17961, with plasmid sequences, and a high-quality (>98% complete) build for A. baumannii strain AB09-003. These genome sequences were generated by combining short-read Illumina and long-read Oxford Nanopore MinION sequencing data using the Unicycler hybrid assembly pipeline.

## ANNOUNCEMENT

Acinetobacter baumannii is a Gram-negative, opportunistic bacterial pathogen that primarily causes infections in health care settings (1). This organism has become a top priority for the development of new treatments due to extremely high rates of antibiotic resistance worldwide (2). Comparing genome sequence data from both historic and current A. baumannii isolates may provide new insights into the emergence and increased antibiotic resistance of this pathogen. Here, we report the genome sequences of A. baumannii strain ATCC 17961 (CDC7788), a clinical isolate collected prior to 1964 (3, 4), and A. baumannii strain AB09-003, a military isolate collected from the cerebrospinal fluid of a wounded soldier and received at the University of Miami (Miami, FL) in 2009.

For DNA isolation, bacteria were grown in lysogeny broth (LB, Lennox formulation) for approximately 16 h at 37°C. DNA for short-read sequencing was purified through extractions with Tris-buffered phenol, 1:1 phenol-chloroform, and cetyltrimethylammonium bromide (CTAB) (5). Paired-end sequencing libraries were prepared with a TruSeq DNA library prep kit (Illumina) and were sequenced using the Illumina MiSeq system with v3 chemistry (2 × 300 bp) by the Genomic Sciences Laboratory at North Carolina State University, Raleigh, NC. Adapter sequences were removed using Trimmomatic v0.32 (6), and the reads were quality filtered and trimmed using the following settings: LEADING:5, SLIDINGWINDOW:4:15, CROP:295, MINLEN:40. The resulting Illumina data sets contained 1,609,266 read pairs with an average length of 293 bp (approximately 943 Mbp) for strain ATCC 17961 and 1,665,932 read pairs with an average length of 293 bp (approximately 976 Mbp) for strain AB09-003. DNA was isolated for MinION sequencing using a Wizard genomic DNA isolation kit (Promega). Sequencing libraries were prepared using a rapid sequencing kit (SQK-RAD004; Oxford Nanopore Technologies) and sequenced using a SpotON flow cell (R9.4) and a MinION sequencing device. Data acquisition was performed using MinKNOW v3.1.8 software, base calling was done using the Oxford Nanopore base caller Guppy v2.1.3 with default settings, and adapter sequences were removed using Porechop v0.2.4 with default settings (https://github.com/rrwick/Porechop). The final long-read data sets contained 74,740 reads at an average length of 6,799 bp for strain ATCC 17961 and 19,496 reads at an average length of 6,720 bp for strain AB09-003. Genome sequences for both strains were generated by combining data from both the Illumina and MinION data sets using Unicycler v0.4.3 (7), as described previously (8). After genome assembly, the contaminating sequences for Phi X174, a control for Illumina sequencing (9), were collapsed into a single contig that was removed from the final assembly for each strain. The completed genome sequences were annotated using the NCBI Prokaryotic Genome Annotation Pipeline v5.0 (10).

For A. baumannii strain ATCC 17961, these procedures resulted in a fully resolved genome sequence consisting of a single circular chromosome of 4,016,386 bp and two plasmids, pAB17961-1 (9,395 bp) and pAB17961-2 (6,667 bp). The G+C content was 39.1%. For A. baumannii strain AB09-003, the resulting assembly consisted of 17 contigs with a total length of 3,952,207 bp (*N*_50_, 3,902,515) and a G+C content of 39.1%. The majority of the sequence (98.7%) is present in a single contig of 3,902,513 bp, and the remaining 16 contigs contain approximately 50 kb that could not be fully resolved due to multiple repetitive genetic elements.

### Data availability.

The completed genome sequences and raw sequence data for A. baumannii strain ATCC 17961 are available through NCBI under BioProject accession no. PRJNA437968, GenBank accession no. CP065432 (chromosome sequence), CP065433 (plasmid pAB17961-1), and CP065434 (plasmid pAB17961-2), and Sequence Read Archive (SRA) accession no. SRX3783012 (Illumina raw sequence data) and SRX9401252 (MinION raw sequence data). The data for A. baumannii strain AB09-003 are available under BioProject accession no. PRJNA615908, GenBank accession no. JADPLY000000000 (genome sequence), and SRA accession no. SRX8013862 (Illumina raw sequence data) and SRX9408185 (MinION raw sequence data).
